# Ki67 expression in invasive breast cancer: the use of tissue microarrays compared with whole tissue sections

**DOI:** 10.1007/s10549-017-4270-0

**Published:** 2017-05-06

**Authors:** Abir A. Muftah, Mohammed A. Aleskandarany, Methaq M. Al-kaabi, Sultan N. Sonbul, Maria Diez-Rodriguez, Chris C. Nolan, Carlos Caldas, Ian O. Ellis, Emad A. Rakha, Andrew R. Green

**Affiliations:** 1 Department of Histopathology, Division of Cancer and Stem Cells, School of Medicine, Nottingham City Hospital, The University of Nottingham and Nottingham University Hospitals NHS Trust, Nottingham, UK; 2 Department of Pathology, Faculty of Medicine, University of Benghazi, Benghazi, Libya; 3 Department of Pathology, College of Medicine, Al Mustansiriyah University, Baghdad, Iraq; 4 Cancer Research UK Cambridge Institute, Li Ka Shing Centre, University of Cambridge, Cambridge, UK

**Keywords:** Ki67, Breast Cancer, Immunohistochemistry, Prognosis, METABRIC

## Abstract

**Background:**

Although the prognostic value of Ki67 in breast cancer is well documented, using optimal cut-points for patient stratification, reproducibility of the scoring and interpretation of the results remains a matter of debate particularly when using tissue microarrays (TMAs). This study aims to assess Ki67 expression assessed on TMAs and their matched whole tissue sections (WTS). Moreover, whether the cut-off used for WTS is reproducible on TMA in BC molecular classes and the association between Ki67 expression cut-off, assessed on TMAs and WTS, and clinicopathological parameters and patient outcome were tested.

**Method:**

A large series (*n* = 707) of primary invasive breast tumours were immunostained for Ki67 using both TMA and WTS and assessed as percentage staining and correlated with each other, clinicopathological parameters and patient outcome. In addition, *MKI67* mRNA expression was correlated with Ki67 protein levels on WTS and TMAs in a subset of cases included in the METABRIC study.

**Results:**

There was moderate concordance in Ki67 expression between WTS and TMA when analysed as a continuous variable (Intraclass correlation coefficient = 0.61) and low concordance when dichotomised (kappa value = 0.3). TMA showed low levels of Ki67 with mean percentage of expression of 35 and 22% on WTS and TMA, respectively. *MKI67* mRNA expression was significantly correlated with protein expression determined on WTS (Spearman Correlation, *r* = 0.52) and to a lesser extent on TMA (*r* = 0.34) (*p* < 0.001). Regarding prediction of patient outcome, statistically significant differences were detected upon stratification of patients with tumours expressing Ki67 at 10, 15, 20, 25 or 30% in TMA. Using TMA, ≥20% Ki67 provided the best prognostic cut-off particularly in triple-negative and HER2-positive classes.

**Conclusion:**

Ki67 expression in breast cancer can be evaluated using TMA although different cut-points are required to emulate results from WTS. A cut-off of ≥20% for Ki67 expression in BC provides the best prognostic correlations when TMAs are used.

**Electronic supplementary material:**

The online version of this article (doi:10.1007/s10549-017-4270-0) contains supplementary material, which is available to authorized users.

## Introduction

Ki67 has been extensively assessed and reported as a prognostic and predictive marker in invasive breast cancer (BC) [1–7]. High Ki67 expression in BC is associated with worse prognosis. In two meta-analyses published in 2007 and 2008, high Ki67 expression in both node-positive and node-negative invasive BC showed significantly worse overall and disease-free survival [8, 9]. Additionally, results of a systematic review support the role of Ki67 as a prognostic marker [10] and as an independent predictive factor for neoadjuvant chemotherapy in BC patients [5, 6, 11]. Furthermore, the St. Gallen consensus panel has recommended Ki67 as a marker for the definition of intrinsic BC subtypes to differentiate between luminal A and luminal B subgroups [12, 13].

In clinical practice, the evaluation of prognostic/predictive factors usually depends upon the stratification of the patients into distinct risk groups based on the status of such factor. The common approach is the choice of an optimal cut-off point for the prognostic/predictive factor, assessed as a continuous variable, e.g. percentage of cells stained, to define these groups. The optimal cut-point for Ki67 in BC is currently debatable despite the large number of published studies reporting significant results [14, 15]. The recent report on the second phase of the Ki67 trial reported that there was a need to standardise the pre-analytical and analytical features for Ki67 immunohistochemistry, so that it can be incorporated to drive patient-care decisions in clinical practice [16]. In 2009, the St. Gallen panel proposed that Ki67 expression should be stratified into three groups: low <15%, intermediate 16–30% and high >30% [17]. This was based on univariate analysis carried out with different Ki67 expression cut-points to find those best stratifying the patients with lowest significant *p* values according to survival using Ki67 immunoreactivity and standardised mitotic index [18]. In 2011, St. Gallen recommended an alternative KI67 cut-point at 14% in order to separate Oestrogen Receptor (ER)-positive tumours into luminal A (<14%) and luminal B (≥14%) [12]. This was derived from comparison with gene array data as a prognostic factor [19]. In 2013, St Gallen revised their threshold to ≥20% for ‘high’ Ki67 status with the option to also use locally specified cut-points [13]. Recently, at the 2015 St. Gallen Breast Cancer Conference, a median cut-off value of Ki67 within the range of 20–29% to differentiate ‘luminal B-like’ has been recommended [20]. As shown by Urruticoechea et al. [21], up to 17 studies that included more than 200 patients displayed statistically significant association between Ki67 and prognosis given that convincing evidence for a biological relationship. However, the cut-offs to discriminate a high from low level of Ki67 varied from 1 to 29%, consequently limiting its clinical utility. Furthermore, during the past decade, multiple research studies have additionally reported the assessment of Ki67 in BC using tissue microarrays (TMA) platform [14] [15], although it remains unclear as to their validity and comparison with assessment in whole tissue sections (WTS).

In this study, we aim to assess BC proliferative fraction using Ki67 assessment utilising matched cases prepared as TMA and WTS taking into account the optimal cut-off value for Ki67 assessed on TMA, the common method of proliferation assessment in the research setting on large cohorts. Herein we aimed at determining (1) to what extent Ki67 protein as well as transcriptome levels are matched between TMA and WTS; (2) whether the cut-point used for WTS is reproducible using TMA in different molecular classes. For the latter aim, the association between Ki67 expression cut-points assessed on TMAs and WTS and the standard clinicopathological variables and patient outcome was tested as endpoints.

## Materials and methods

### Patient cohort

This study was approved by the Nottingham Research Ethics Committee 2 under the title ‘Development of a molecular genetic classification of breast cancer’.

The expression of Ki67 was assessed on 707 cases of invasive BC cases using WTS and TMA. TMAs were prepared using 0.6-mm cores sampled from the invasive tumour edge as previously described [22]. Cases were derived from the retrospective Nottingham Tenovus Primary Breast Carcinoma Series. This is a consecutive well-defined series of early-stage primary operable invasive BC (TNM Stage I–III, excluding T3 and T4 tumours) from patients presented to Nottingham City Hospital from 1988 to 1998. The age of the patients was ≤70 years (Supplementary Table 1). Moreover, the clinical details of the patients including age and menopausal status as well as the tumour details including tumour size, grade, lymphovascular invasion (LVI) and lymph node status were also available and prospectively maintained. Survival data include Breast Cancer-Specific Survival (BCSS), in months, from the date the primary surgical treatment to the time of death from breast cancer. Molecular classes were defined as luminal (ER+ and/or PR+), HER2+ (HER2+ regardless of the expression of other markers) and triple-negative (TN; HER2−, ER− and PR−). In this cohort, transcriptomic data for MKI67 were available for a subset (*n* = 101) from Nottingham cases that were included in the METABRIC cohort [23].

### Immunohistochemistry

4-μm sections were freshly cut from representative paraffin blocks and transferred onto slides (Surgipath Xtra Adhesive, Leica, Germany). Slides were incubated on a 60 °C hotplate for 10 min, followed by deparaffinisation and rehydration using xylene and graded alcohol. For antigen retrieval, sections were incubated in Citrate Buffer at pH 6.0 for 20 min using microwave. Manual immunohistochemistry staining was performed using either the Novolink™ Max Polymer Detection Kit (Leica, Newcastle, UK) for the TMAs and the standard streptavidin–biotin complex method for the WTS following manufacturer’s instructions and as previously described [4]. Optimised primary antibody, MIB-1 monoclonal mouse diluted 1:100 (Dako, Ref-M7240) antibody was applied and incubated for 1 h at room temperature. Finally, DAB chromogen reagent was incubated for 5 min, then 0.1% Haematoxylin was added as a counter stain. Dehydration, clearing, mounting and cover-slipping were performed as previously described. Human tonsil sections were used as a positive control, while negative controls were performed by omitting the application of primary antibody.

### Ki67 assessment

Ki67-stained TMA slides were scanned into high-resolution digital images (0.45 µm/pixel) using a NanoZoomer slide scanner (Hamamatsu Photonics Welwyn Garden City, UK). Scoring of TMA was performed on digital images using a web-based interface (Distiller, Slidepath Ltd., Dublin, Ireland). Only the invasive breast cancer cells present in the TMA cores were assessed for Ki67 staining and scored as a percentage of the positively stained nuclei [15]. All tumour cell nuclei with homogenous granular staining, multiple speckled staining or nucleolar staining were regarded as positively stained regardless of their staining intensity [24]. To test for inter-observer concordance, three TMA slides (*n* = 350) were re-scored by another observer (MA). Scoring of WTS was performed in the areas with highest number of positive nuclei (hot spot) within the invasive component of the tumour as previously described [4]. Hot spots were identified by scanning the section for immunostaining evaluation using a light microscope at low power magnification (×100). Ki67 was expressed as the percentage of positive malignant cells in 1000 malignant cells assessed under high power magnification (×400). To assess for inter-observer concordance, a subset of cases (*n* = 180) was re-scored by another observer (AM).

### Statistical analysis

Statistical analysis was performed using IBM SPSS software version 22 (SPSS Inc., Chicago, IL, USA). For all statistical tests a *p* value < 0.05 was considered significant. Spearman correlation test, Intraclass Correlation Coefficient (ICC) and kappa statistic were used to test the reproducibility and the correlation between the Ki67 assessment between TMA and WTS. In kappa, complete agreement is reflected by a value of 1.0 and only by chance alone results in a value of zero. Although in the literature there is no agreed standard criteria for kappa value that indicates adequate agreement, Landis and Koch proposed the following agreement measures for categorical data: kappa <0.00 represents poor agreement, 0.00–0.20 slight, 0.21–0.40 fair, 0.41–0.60 moderate, 0.61–0.80 substantial and 0.81–1.00 almost perfect agreement [25]. Accordingly, Mikami et al. suggested that based on the similarity to the kappa coefficient, ICC between 0.41 and 0.60 is considered as moderate correlation; 0.61–0.80 as substantial correlation and >0.80 as a perfect correlation [26]. Chi-square, Kaplan–Meier and Cox regression tests were applied to test the association with the standard clinicopathological parameters, other prognostic biomarkers and outcome of breast cancer patients. This study adheres to REporting recommendations for tumour MARKer prognostic studies (REMARK) criteria [27].

## Results

### Comparison of Ki67 expression between TMA and WTS (Protein expression)

Using WTS, Ki67 expression was not normally distributed (Supplementary Fig. 1A; range 0–99%): the mean percentage was 34.8%, while the median percentage was 20%. Similar to the distribution seen with WTS, Ki67 expression scored on TMAs was not normally distributed (Supplementary Fig. 1B; range 0–95%): the mean percentage was 21.9%, while the median was 10%. The reproducibility of Ki67 assessment on TMAs showed that there is a significant correlation (*p* < 0.001) between the two observers’ scoring. Agreement between the two observers showed an almost perfect concordance (*p* < 0.001) as tested by ICC intraclass correlation coefficient (ICC = 0.870, 95% confidence interval (CI) = 0.838–0.896). On the other hand, Ki67 scoring on WTS showed substantial concordance (ICC = 0.75, 95% CI = 0.670–0.815).

**Fig. 1:**
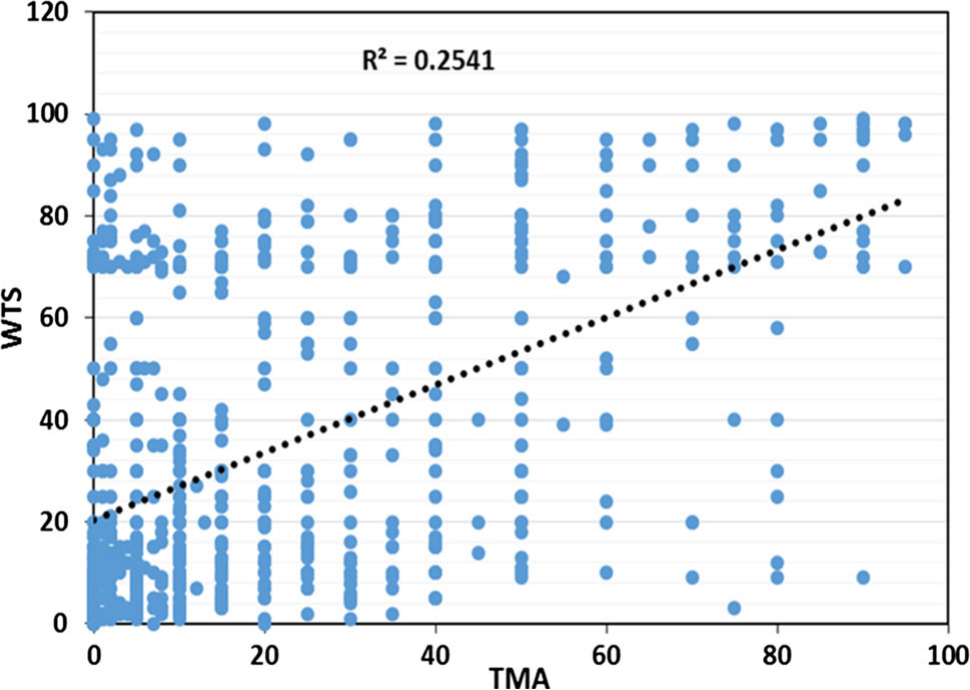
Correlation between Ki67 expressions assessed on matched cases on WTS and TMA

When Ki67 expression was compared between TMA and WTS, there was a significant correlation when measured as a continuous variable (*p* < 0.001), the Spearman’s correlations 0.50 with an *r* ^2^ value of 0.025 (Fig. 1). When ICC was used, substantial correlation was observed (ICC = 0.61, *p* < 0.001, 95% CI = 0.45–0.71).

To evaluate the reproducibility of Ki67 expression between WTS and TMA differing cut off points between platforms were assessed. The highest concordance was obtained when WTS is 10%. Therefore, for further analysis, the data were dichotomised at 10% as a fixed cut-point for WTS, which was published previously as the optimal cut-off [28] as well as, and at different cut-points for TMA to evaluate the reproducibility of the WTS Ki67 expression: 5, 15, 20, 25 and 30% (Table 1). As shown in Supplementary Fig. 2, higher cut-off values resulted in misclassification of a higher percentage (62.8%) of cases assessed on TMAs into the low proliferation group compared with their matched WTS. Conversely, lower cut-off values resulted in a higher number of cases matching between the positive cases. However, there was a high percentage (49.8%) of false-positive cases when assessed on TMA compared with WTS. A cut-off of 20% for Ki67 expression determined using TMAs seems to give the highest concordance in both positive and negative groups with less both false-positive and false-negative Ki67 expressions at 10% determined using WTS.

**Table 1:** Results of classification of the studied cases at different Ki67 cut-off points (on TMAs) as compared with WTS (10% cut-off)

Ki67 on TMAs	Ki67 on WTS (at 10%)	
	Negative	Positive
5% low High	102 (50.2)	91 (18.1)
	101 (49.8)	413 (81.9)
10% low High	135 (47.9)	147 (52.1)
	68 (16.0)	357 (84.0)
15% low High	157 (43.9)	201 (56.1)
	46 (13.2)	303 (86.8)
20% low High	170 (41.8)	237 (58.2)
	33 (11.0)	267 (89.0)
25% low High	177 (39.1)	276 (60.9)
	26 (10.2)	228 (89.8)
30% low High	181 (37.2)	305 (62.8)
	22 (10.0)	199 (90.0)

**Fig. 2:**
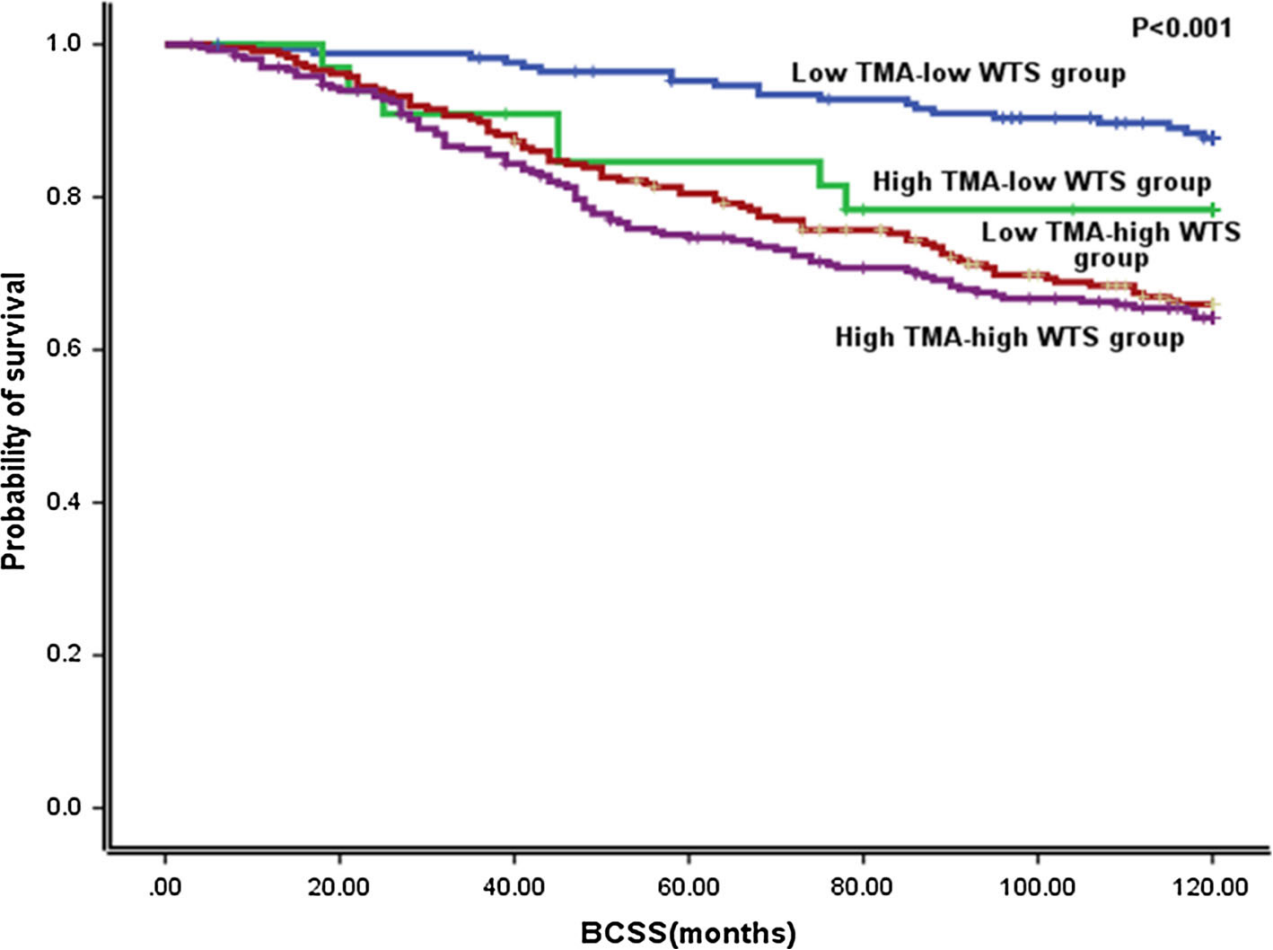
BCSS for Ki67 high/low groups as defined by Ki67 expression on WTS and TMA at 10 and 20%, respectively

To test for the impact of cut-points on patient outcome as an end point, cases were classified based on their Ki67 expression, whether it was low or high, on TMAs and matched WTS. Therefore, using 10% as a cut-off for Ki67 on WTS and 20% as a cut-off for on TMAs, four groups were produced. Group one comprised cases with low Ki67 expression on TMAs and their matched WTS, group two comprised cases with high Ki67 expression on TMAs with low Ki67 expression on their matched WTS, group three cases with low Ki67 expression on TMAs with high Ki67 expression on their matched WTS and group four with high Ki67 expression on TMAs with high Ki67 expression on their matched WTS. Statistically significant differences were observed between these groups regarding patients outcome (Long Rank (LR) = 31.79, *p* < 0.001), (Fig. 2). Interestingly, there was no significant difference between the groups including high Ki67 expression on WTS (LR = 0.39, *p* = 0.52); therefore, they seem to have more or less similar poor outcome.

### Comparison of Ki67 expression between TMA and WTS in different molecular subclasses

The studied cases were defined with regard to their molecular class as luminal, which includes ER positive cases; HER2+ and triple-negative (TN), which includes ER−, PR− and HER2− cases. Assessment of the concordance between Ki67 expression on TMAs and WTS in molecular subclasses using different cut-points showed that in TN and HER2+ tumours, a cut-off of 20% seemed to give the highest concordance between WTS and TMAs. In TN classes, the highest concordance between the positive cases (91.5%) with the lowest number of false positives (8.5%) was shown with 20% cut-point using TMA (Supplementary Fig. 3). Similarly, for HER2-positive tumours 20% was the optimal cut-off to classify tumour proliferation using TMAs where there was 96.2% concordance for the positive cases and 3.8% false-positive cases (Supplementary Fig. 4). However, in the luminal class, there was no optimal cut-off for Ki67 determined on TMAs which was reproducible to the Ki67 scoring on WTS (Supplementary Fig. 5).

### Comparison of Ki67 expression on TMA and WTS with *MKI67* mRNA expression

**Fig. 3:**
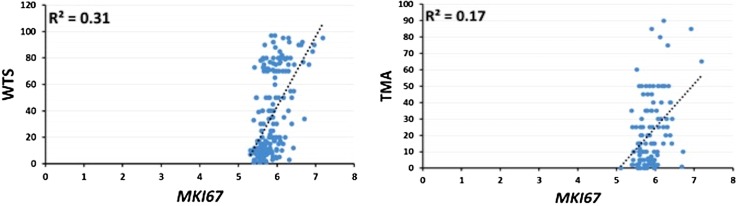
Correlation between *MKI67* mRNA and Ki67 assessed on WTS and TMA

Using the METABRIC cohort, *MKI67* mRNA data were available for 197 and 123 cases matched with WTS and TMA cases, respectively. The correlation between *MKI67* mRNA and Ki67 protein expression determined using 197 cases of WTS was significant, *p* < 0.001 and Spearman’s correlation coefficient = 0.587. Although the correlation between *MKI67* mRNA and 123 cases Ki67 assessed on TMAs was significant (*p* < 0.001), Spearman’s correlation was less compared with WTS = 0.343. Figure 3 shows the correlation between *MKI67* mRNA with WTS (*r* ^2^ = 0.31) and TMA (*r* ^2^ = 0.17). In matching 101 BC cases assessed on both TMA and WTS, *MKI67* mRNA expression was evaluated. Higher significance was observed for Ki67 assessed on WTS (Spearman’s correlation coefficient was 0.529 and *p* < 0.001) than on TMA (Spearman’s correlation coefficient was 0.341 and *p* < 0.001).

### Ki67 and clinicopathological variables and patient outcome

**Fig. 4:**
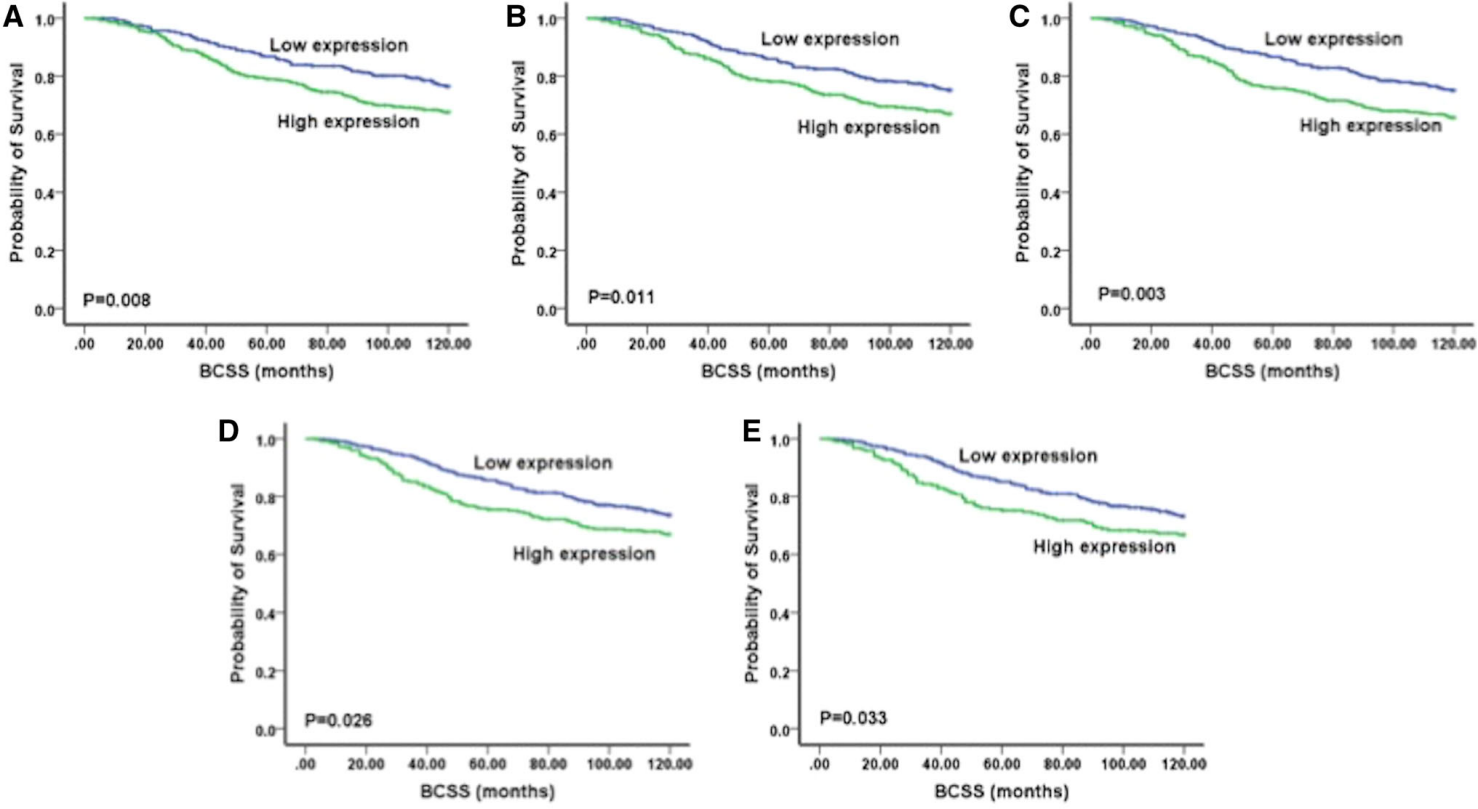
Kaplan Meier plots illustrating BCSS for different Ki67 expression cut-offs assessed on TMAs: **a** 10%, **b** 15%, **c** 20%, **d** 25% and **e** 30%

When assessing Ki67 expression on TMA cores, high expression of Ki67 (>20%) was significantly associated with larger tumour size, higher grade, more nuclear pleomorphism, higher mitotic scores and less tubule formation (Supplementary Tables 2 and 3, *p* < 0.05). Regarding patient outcome, univariate survival analysis of Ki67 determined using TMAs showed that a cut-off of 20% was the most significantly associated with BCSS (LR = 8.76, *p* = 0.003, Fig. 4). Furthermore, using Cox regression analysis, different cut-offs points and the relations with BCSS were investigated (Table 2). Interestingly, 20% showed the highest risk on patients’ survival (hazards ratio, HR = 1.52, 95% CI = 1.15–2.0, *p* = 0.003). On the other hand, univariate survival analysis of Ki67 on WTS using Kaplan–Meier test showed that Ki67 at a cut-off of 10% was significantly associated with BCSS (LR = 30.1, *p* < 0.001). Furthermore, using Cox regression, >10% expression of Ki67 gave the highest risk on patients’ survival (HR = 2.95, 95% CI = 1.96–4.43, *p* < 0.001; Table 3).

**Table 2:** Univariate Cox regression analysis for predictors of BCSS at different Ki67 cut-offs points assessed on TMA

Ki67 cut-offs (%)	*p* value	HR	95% CI
10	0.008	1.496	1.111–2.016
15	0.011	1.440	1.086–1.908
20	0.003	1.519	1.149–2.009
25	0.027	1.379	1.038–1.832
30	0.034	1.370	1.024–1.832

**Table 3:** Univariate Cox regression analysis for predictors of BCSS at different Ki67 cut-offs points assessed on WTS

Ki67 cut-offs (%)	*p* value	HR	95% CI
10	<0.001	2.953	1.967–4.432
15	<0.001	1.984	1.451–2.714
20	<0.001	2.050	1.521–2.763
25	<0.001	1.970	1.479–2.623
30	<0.001	1.843	1.392–2.442

## Discussion

One of the attractive alternatives for using WTS section in the research field is the use of the TMAs since a large number of the tissue samples can be simultaneously analysed under the same experimental conditions. Additionally, it is a time, resource and cost effective [29, 30]. There is a mounting evidence indicating the usefulness TMAs in translational biomarker discovery/validation studies utilising materials from large scale population-based studies showing high concordance rates between TMA and WTS [31]. However, it is imperative to recognise its limitations especially in interpreting the results of biomarkers with considerable spatial intra-tumour heterogeneity of expression. In this study, our results assess different aspects regarding the comparison between the WTS and TMA demonstrating its relation to the reproducibility. There is a significant concordance between the Ki67 expression in the WTS and TMA. Importantly, concordance was substantial when continuous data are used (i.e. Ki67%) and much lower when dichotomised. The latter observation is probably due to the more tendency of TMA to give lower Ki67 estimates than the whole sections, which we observed in Ki67 scores of matched cases assessed on WTS and TMAs. In addition to the intratumoral heterogeneity, this is could be as a result of using one TMA core. Using more than one TMA core or a larger core diameter has been suggested to achieve better representation of the tumour proliferative fraction. Although Karlsson et al. [32] and Batistatou et al. [33] showed excellent agreement between TMAs and whole sections they have used only 10 and 88 cases of BC, respectively.

To assess the inter-observer reproducibility of Ki67 assessment, a significant correlation between the scorers was observed in Ki67 using TMAs. This result is consistent with results published by an international Ki67 reproducibility study which showed a high intra-laboratory reproducibility [15]. However, the same study resulted in only moderate reproducibility between different laboratories, which necessitates a standardised scoring methodology. The comparison between the two Ki67 assessed on WTS and TMA in relation to the clinicopathological parameters and BC-related biomarkers yielded comparably similar associations. As expected, high Ki67 was significantly associated with larger tumour size, higher tumour grade, more nuclear pleomorphism and mitotic scores.

Currently, consensus is lacking regarding an optimal cut-off for Ki67 expression both in the clinical setting and research settings. This affected the comparison of Ki67 expression in different clinical trials [15]. Ki67 has a significant prognostic value over a wide range of cut-offs and the optimisation of one cut-off is controversial. For instance, Urruticoechea et al. demonstrated, after evaluating of 18 studies, the wide range of Ki67 cut-points ranging from 1% to up to 29%. Accordingly, they concluded that this varied Ki67 cut-off may be the reason for its restricted clinical use [21]. One possible explanation for the wide range of cut-offs could be the absence of standardisation in the pre-analytical tissue handling, in terms of duration of ischaemia, time to fixation, dilution and pH of formalin used in tissue fixation and procedures of antigen retrieval which largely depend on the pre-analytical phase. Pathologist’s scoring of the immunohistochemical staining also has a minor role [34]. Therefore, standardised approaches in the pre-analytical tissue handling, especially adequate fixation, are crucial for reliable proliferative fraction assay.

In the current study, we evaluated a wide range of cut-points in the studied series, and all were significant with patient outcome as the study end point. The same cut-off points (10, 15, 20, 25 and 30%) have been examined in the comparison of Ki67 on WTS and all gave significant results with BCSS. Interestingly, there are different cut-offs that have the best correlation with clinicopathological parameters, biomarkers and patients outcome according to the type of tissue used, WTS or TMA. 10% seems to be the best cut-off when the WTS was used, while the statistical significance was higher using 20% as cut-off when Ki67 was assessed on TMAs. This cut-off of 10% was previously used in several series published by others for different purposes. For instance, Pathmanathan et al. evaluated the utility of Ki67 as a prognostic marker in a series of patients and emphasised that the highest sensitivity and specificity of Ki67 cut-off is 10% after evaluation of different cut-offs using 203 cases as WTS [35]. Furthermore, Shui et al. using BC cases processed as WTS concluded that assessment of Ki67 at 10% is a candidate for a standard method in breast cancer clinical practice [36]. Importantly and supporting to our results suing TMAs, the St Gallen has revised the threshold for ‘high’ Ki67 status to ≥20% with the option to also use locally specified cut-points [13].

## Conclusions

Ki67 expression can be evaluated using WTS and TMA; however, due to the reported substantial heterogeneity of Ki67 expression in BC the latter should be interpreted with caution. Assessment of Ki67 as a continuous variable may better reflect the proliferative status than the predefined dichotomised values currently in use. A cut-point of 20% in BC when assessing Ki67 on TMAs appears to be optimum both at concordance with WTS as well as with patients’ outcome.

## Electronic supplementary material

Below is the link to the electronic supplementary material.

Supplementary Fig. 1: Shows the distribution of Ki67 expression on: A) WTS, and B) on TMAs. (TIFF 67 kb)

Supplementary Fig. 2: Shows the percentage of cases classified as low/high proliferative assessed on TMAs and WTS using the whole cohort (*n* = 707). (JPEG 788 kb)

Supplementary Fig. 3: Shows the percentage of matched cases between TMA and WTS in the triple negative BC subtype. (TIFF 93 kb)

Supplementary Fig. 4: Shows the percentage of matched cases between TMA and WTS in the HER2 positive BC subtype. (TIFF 90 kb)

Supplementary Fig. 5: Shows the percentage of matched cases between TMA and WTS in the luminal BC subtype. (TIFF 120 kb)

Supplementary material 6 (DOCX 19 kb)

Supplementary material 7 (DOCX 18 kb)

Supplementary material 8 (DOCX 17 kb)
