# Supplementary figures and images for: Ki67 expression in invasive breast cancer: the use of tissue microarrays compared with whole tissue sections
Source: Breast Cancer Res Treat. 2017 May 6;164(2):341–8. doi: 10.1007/s10549-017-4270-0 (PMC5487701; doi:10.1007/s10549-017-4270-0)

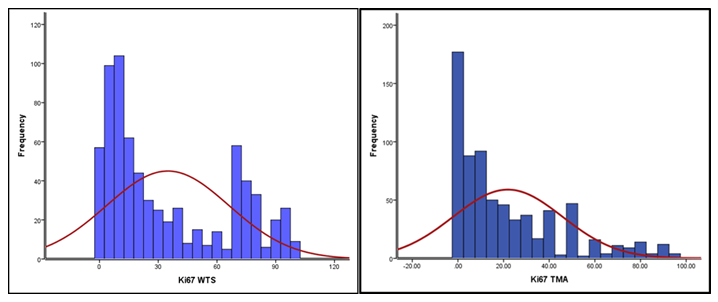

Supplement: Supplementary file 1 — Supplementary Fig. 1: Shows the distribution of Ki67 expression on: A) WTS, and B) on TMAs. (TIFF 67 kb) [file 10549_2017_4270_MOESM1_ESM.tif]

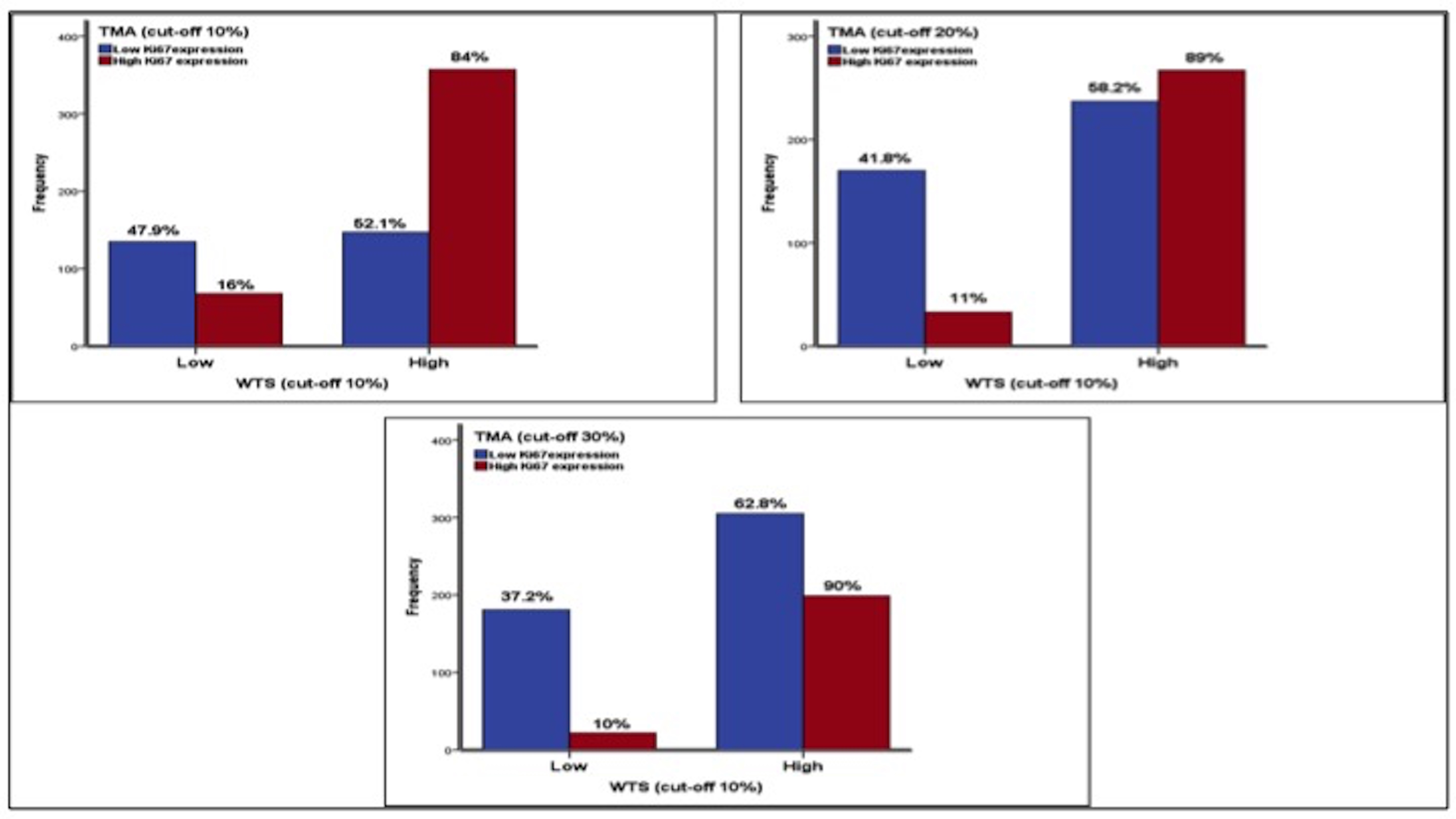

Supplement: Supplementary file 2 — Supplementary Fig. 2: Shows the percentage of cases classified as low/high proliferative assessed on TMAs and WTS using the whole cohort (n = 707). (JPEG 788 kb) [file 10549_2017_4270_MOESM2_ESM.jpg]

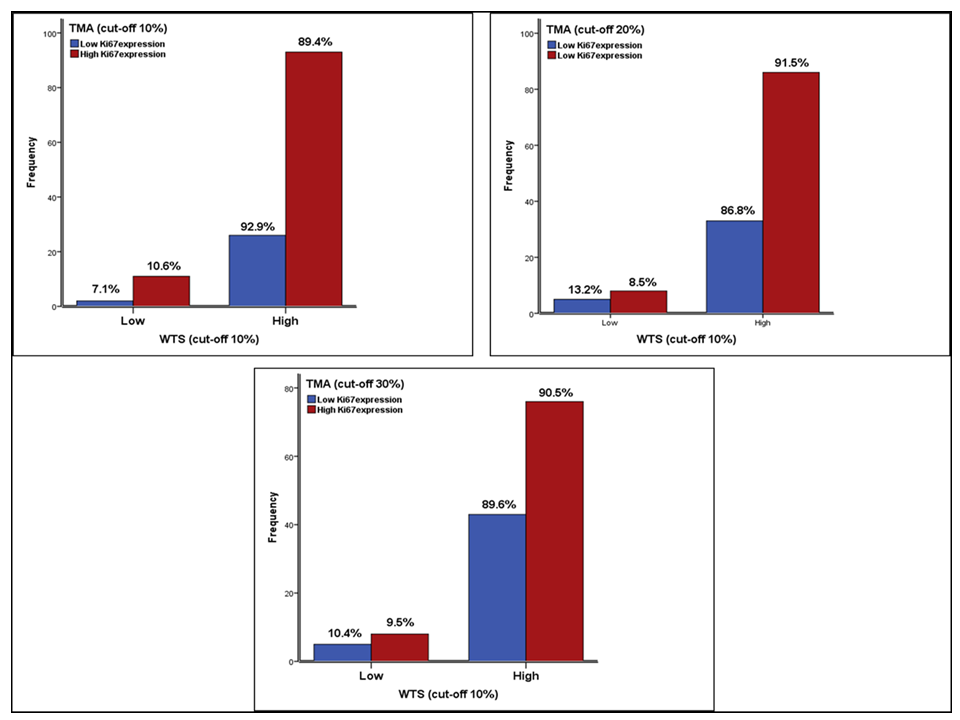

Supplement: Supplementary file 3 — Supplementary Fig. 3: Shows the percentage of matched cases between TMA and WTS in the triple negative BC subtype. (TIFF 93 kb) [file 10549_2017_4270_MOESM3_ESM.tif]

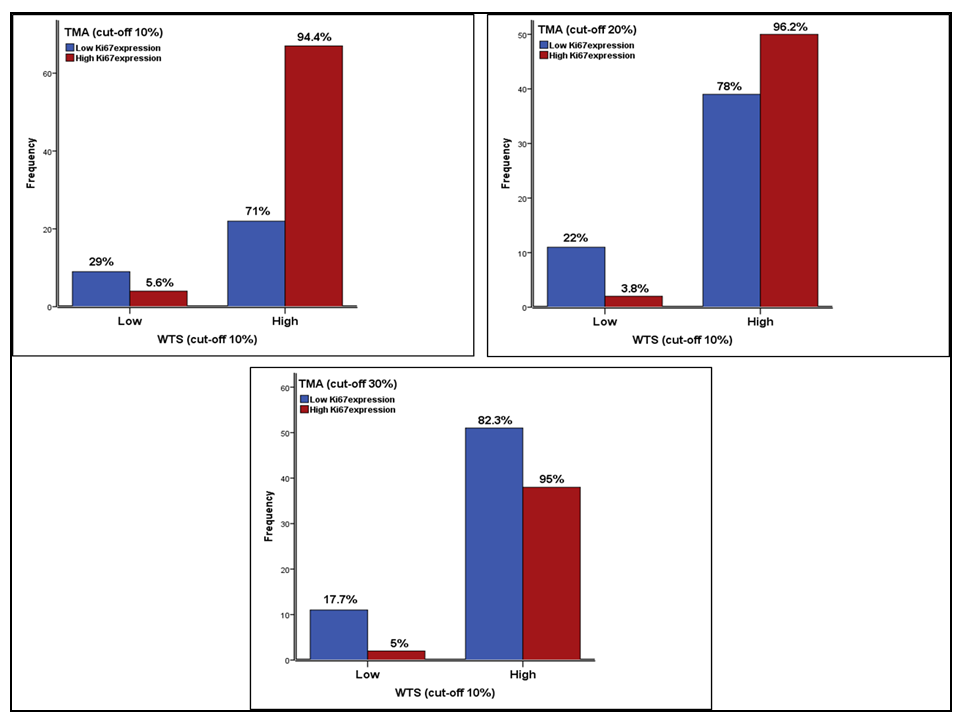

Supplement: Supplementary file 4 — Supplementary Fig. 4: Shows the percentage of matched cases between TMA and WTS in the HER2 positive BC subtype. (TIFF 90 kb) [file 10549_2017_4270_MOESM4_ESM.tif]

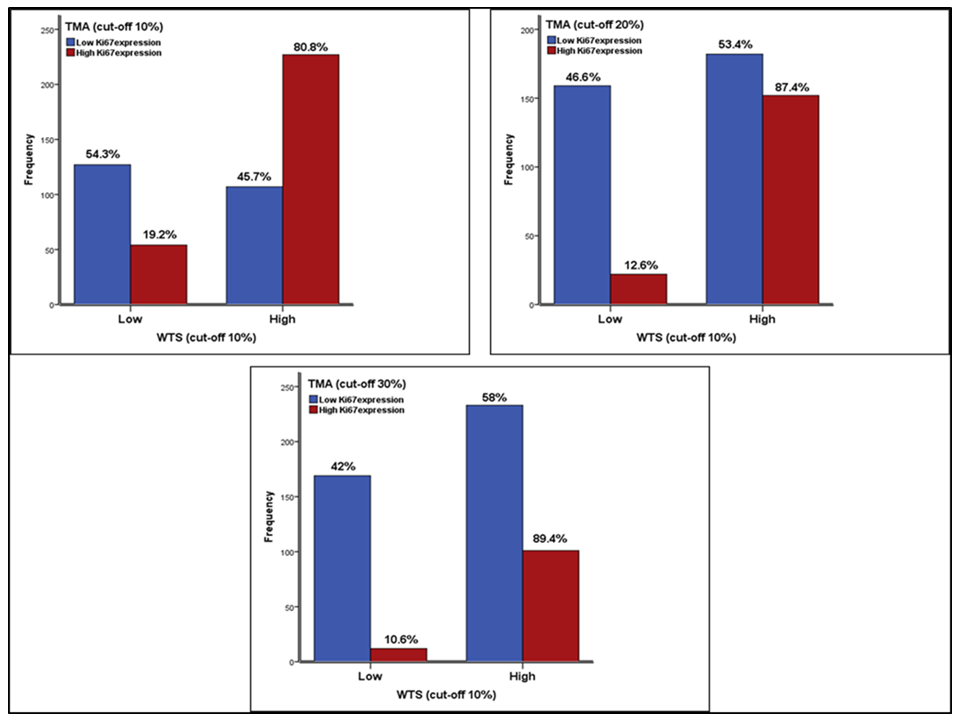

Supplement: Supplementary file 5 — Supplementary Fig. 5: Shows the percentage of matched cases between TMA and WTS in the luminal BC subtype. (TIFF 120 kb) [file 10549_2017_4270_MOESM5_ESM.tif]
