# Supplementary material for: Ki67 expression in invasive breast cancer: the use of tissue microarrays compared with whole tissue sections
Source: Breast Cancer Res Treat. 2017 May 6;164(2):341–8. doi: 10.1007/s10549-017-4270-0 (PMC5487701; doi:10.1007/s10549-017-4270-0)
Supplement: Supplementary file 6 — Supplementary material 6 (DOCX 19 kb) [file 10549_2017_4270_MOESM6_ESM.docx]

| Parameter | Number (%) |
| --- | --- |
| Patient Age  ≤ 50 years  > 50 years | 271 (38.4)  436 (61.6) |
| Menopausal Status  Premenopausal  Postmenopausal | 283 (40.1)  423 (59.9) |
| Tumour Grade  1  2  3 | 93 (13.2)  216 (30.6)  397 (56.2) |
| Pleomorphisms  1  2  3 | 10 (1.5)  233 (33.9)  444 (64.6) |
| Tubule Formation  1  2  3 | 34 (4.9)  211 (30.7)  443 (64.4) |
| Mitotic Figures  1  2  3 | 203 (29.5)  132 (19.2)  353 (51.3) |
| Tumour Size  ≤ 2 cm  > 2 cm | 407 (57.6)  300 (42.4) |
| LN status  1 (0 Positive nodes)  2 (1-3 Positive nodes)  3 (>3 Positive nodes) | 405 (57.3)  235 (33.2)  67 (9.5) |
| Nottingham Prognostic Index (NPI)  Good NPI (<3.4)  Moderate NPI (3.41-5.4)  Poor NPI (≥5.4) | 179 (25.3)  389 (55.0)  139 (19.7) |
| Histological tumour type  Ductal No Special Type  Tubular Mixed  Medullary  Lobular Carcinoma  Special types of excellent prognosis*  Mixed NST and Lobular  Mixed NST and other Special Type | 442 (63.1)  113 (16.1)  16 (2.3)  58 (8.3)  25 (3.6)  27 (3.9)  19 (2.7) |
| LVI Negative  Definite | 444 (63.1)  263 (36.9) |
| ER status  ER Negative  ER Positive | 189 (26.8)  516 (73.2) |
| PR status  PR Negative  PR Positive | 291 (42.0)  402 (58.0) |
| HER2 status  HER2 Negative  HER2 Positive | 588 (85.2)  102 (14.8) |
| Molecular Subtype  Luminal  HER2 positive  Triple Negative | 515 (68.8)  102 (13.6)  132 (17.6) |

## Supplementary Table 1: Summary of the characteristics of the study cohort.

* : Invasive Tubular, Invasive Mucinous, invasive Cribriform and Invasive Papillary Carcinomas.
