# Supplementary material for: Ki67 expression in invasive breast cancer: the use of tissue microarrays compared with whole tissue sections
Source: Breast Cancer Res Treat. 2017 May 6;164(2):341–8. doi: 10.1007/s10549-017-4270-0 (PMC5487701; doi:10.1007/s10549-017-4270-0)
Supplement: Supplementary file 7 — Supplementary material 7 (DOCX 18 kb) [file 10549_2017_4270_MOESM7_ESM.docx]

| TMA | Tumour Size (cm) | | | | Grade | | | | |
| --- | --- | --- | --- | --- | --- | --- | --- | --- | --- |
|  | ≤ 2.0 | > 2.0 | p-value | X2 | 1 | 2 | 3 | *p*-value | *χ*^2^ |
| 10 low  high | 154 (54.6) | 128 (45.4) | 0.001 | 11.44 | 69 (24.5) | 114 (40.4) | 99 (35.1) | <0.001 | 97.12 |
|  | 177 (41.6) | 248 (58.4) |  |  | 24 (5.7) | 102 (24.1) | 297 (70.2) |  |  |
| 15 low  high | 192 (53.6) | 166 (46.4) | <0.001 | 13.52 | 80 (22.3) | 142 (39.7) | 136 (38.0) | <0.001 | 108.35 |
|  | 139 (39.8) | 210 (60.2) |  |  | 13 (3.7) | 74 (21.3) | 260 (74.9) |  |  |
| 20 low  high | 218 (53.6) | 189 (46.4) | <0.001 | 17.52 | 84 (20.7) | 165 (40.6) | 157 (38.7) | <0.001 | 124.25 |
|  | 113 (37.7) | 187 (62.3) |  |  | 9 (3.0) | 51 (17.1) | 239 (79.9) |  |  |
| 25 low  high | 238 (52.5) | 215 (47.5) | <0.001 | 16.575 | 88 (19.5) | 175 (38.7) | 189 (41.8) | <0.001 | 110.66 |
|  | 93 (36.6) | 161 (63.4) |  |  | 5 (2.0) | 41 (16.2) | 207 (81.8) |  |  |
| 30 low  high | 255 (52.5) | 231 (47.5) | <0.001 | 19.945 | 89 (18.4) | 186 (38.4) | 210 (43.3) | <0.001 | 107.37 |
|  | 76 (34.4) | 145 (65.6) |  |  | 4 (1.8) | 30 (13.6) | 186 (84.5) |  |  |

## Supplementary Table 2: Association of Ki67 at different cut-off points assessed on TMAs with tumour size and grade of the studied series.
