# Supplementary material for: Ki67 expression in invasive breast cancer: the use of tissue microarrays compared with whole tissue sections
Source: Breast Cancer Res Treat. 2017 May 6;164(2):341–8. doi: 10.1007/s10549-017-4270-0 (PMC5487701; doi:10.1007/s10549-017-4270-0)
Supplement: Supplementary file 8 — Supplementary material 8 (DOCX 17 kb) [file 10549_2017_4270_MOESM8_ESM.docx]

| TMA | Pleomorphism | | | | | Mitosis | | | | |
| --- | --- | --- | --- | --- | --- | --- | --- | --- | --- | --- |
|  | 1 | 2 | 3 | *p*-value | *χ*^2^ | 1 | 2 | 3 | *p*- | *χ*^2^ |
| 10 low  high | 9 (3.3) | 133 (48.7) | 131 (48.0) | <0.001 | 59.23 | 142 (51.8) | 61 (22.3) | 71 (25.9) | <0.001 | 136.35 |
|  | 1 (0.2) | 100 (24.2) | 313 (75.6) |  |  | 61 (14.7) | 71 (17.1) | 282 68.1) |  |  |
| 15 low  high | 9 (2.6) | 161 (46.5) | 176 (50.9) | <0.001 | 59.42 | 169 (48.7) | 76 (21.9) | 102 (29.4) | <0.001 | 155.66 |
|  | 1 (0.3) | 72 (21.1) | 268 (78.6) |  |  | 34 (10.0) | 56 (16.4) | 251 (73.6) |  |  |
| 20 low  high | 9 (2.3) | 180 (45.8) | 204 (51.9) | <0.001 | 65.63 | 182 (46.2) | 89 (22.6) | 123 (31.2) | <0.001 | 165.10 |
|  | 1 (0.3) | 53 (18.0) | 240 (81.6) |  |  | 21 (7.1) | 43 (14.6) | 230 (78.2) |  |  |
| 25 low  high | 9 (2.1) | 191 (43.6) | 238 (54.3) | <0.001 | 56.25 | 187 (42.6) | 97 (22.1) | 155 (35.3) | <0.001 | 136.33 |
|  | 1 (0.4) | 42 (16.9) | 206 (82.7) |  |  | 16 (6.4) | 35 (14.1) | 198 (79.5) |  |  |
| 30 low  high | 10 (2.1) | 200 (42.5) | 261 (55.4) | <0.001 | 56.53 | 193 (40.9) | 106 (22.5) | 173 (36.7) | <0.001 | 137.35 |
|  | 0 (0.0) | 33 (15.3) | 183 (84.7) |  |  | 10 (4.6) | 26 (12.0) | 180 (83.3) |  |  |

## Supplementary Table 3: Association of different Ki67 cut-off points assessed on TMAs with nuclear pleomorphism and mitotic scores of the studied cohort.
